# Peer Review Week 2022: an interview with Franziska Denk and Katryn Stacey about their views on research integrity as scientists and reviewers

**DOI:** 10.1038/s42003-022-03958-w

**Published:** 2022-09-20

## Abstract

In this second Q&A for Peer Review Week, we spoke to Dr. Franziska Denk and Professor Katryn Stacey about their views on research integrity as scientists and reviewers.

**Dr. Franziska Denk is Senior Lecturer at King’s College London.**

**Figure:**
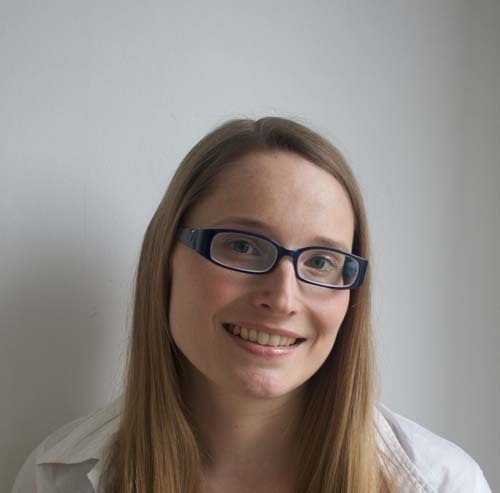
Franziska Denk

**Professor Katryn Stacey is a Professor at the University of Queensland, Australia.**

**Figure:**
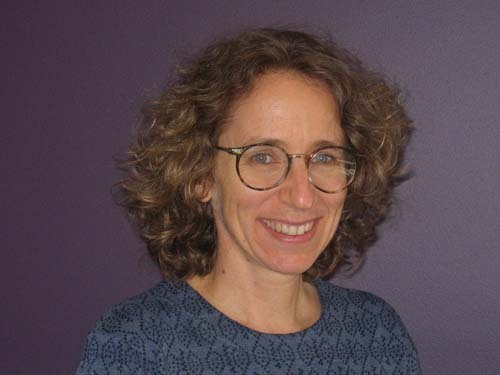
Katryn Stacey

Please tell us about your research interests and current position?

**[FD]:** I’m a Senior Lecturer at King’s College London. My laboratory is investigating various possible causes for how pain might become chronic. We are examining the role of immune cells and other supporting cell types and how they may contribute to making nerves persistently hypersensitive.

**[KS]:** I work in the field of innate immune signalling in response to pathogen products. My PhD work involved trying to transfect plasmid DNA into macrophages, and from the artefacts of that process that I observed, I developed an interest in responses of cells to exogenous DNA. I always try to look closely at the inconvenient results that don’t do what you want, because that is the source of new knowledge. Studying those pesky results led us to demonstrate that macrophages are activated by bacterial DNA, which turns out to be toll-like receptor 9-mediated, and primary macrophages die with cytosolic DNA, which I worked on sporadically over 17 years before identifying the AIM2 inflammasome as the cause. I am now a professor of immunology at the University of Queensland in Australia.

As a researcher, can you share your views on the importance of research integrity and what damage research misconduct can do to the field?

**[FD]:** Personally, I feel that there are two main aspects to this; obviously, outright fraud is always incredibly damaging, not only in terms of its reputational harm, but also in terms of the damage it inflicts on individuals: young scientists get their reputations tangled up in fraud causes, people donating to small research charities have their money misspent, etc. However, I believe that outright fraud is not actually the biggest issue we face. Fraudulent research often raises eyebrows long before it is “publicly” outed and is quickly side-lined within research fields.

I think a much bigger issue is that many studies suffer from fundamental methodological and statistical design flaws that completely invalidate the conclusions that can be drawn from them. Often, the people in charge of these studies are not aware that their work is too poorly executed to further progress. It is easy to be in denial or ignorant about these things – due to discipline-specific silos, entire fields can go through periods of nonsensical study designs.

For example, in neurosciences, it took 3-5 years for everyone to figure out that epigenetic analyses don’t make much sense if we do not work in a tissue-specific manner. We didn’t spend enough time communicating with developmental biologists or cancer researchers who would have told us this. Similarly, many pre-clinical scientists have heard of p-hacking and parrot about power calculations, but simply do not realise that a) many of us have not been taught how to do statistics well and b) that this is an incredibly serious problem. There is a widespread feeling that statistics somehow aren’t all that relevant to basic mechanistic laboratory research.

I think we need to tackle this second issue, using different language that is not scary or moralistic, e.g. by avoiding words like “misconduct” and “integrity”.

**[KS]:** Without a focus on research integrity, a lot of things that are just plain wrong get published. Then time is wasted and the misinformation slows down the rate of scientific advance. The truth will come out in the end, but it might take 5 years or 50 years. Probably the most serious damage done by fraudulent science is the loss of public faith in science. With the massive challenge of climate change facing us at the moment, we need that public trust.

What do you do individually and what practices and values are important in your lab to support integrity?

**[FD]:**In my team, we value sharing data, whether they confirm the null hypothesis or not. We try to make our work accessible, e.g. we build little databases around transcriptomic datasets we produce to maximise their value for the community, e.g. https://rna-seq-browser.herokuapp.com/.

I am very keen to change the narrative of what constitutes great science – away from the idea that it is the result of “individual genius” towards the fact that it is usually the consequence of many people working together in an open and collaborative manner. A good example of this is the team around CAMARADES at the University of Edinburgh (e.g. Emily Sena and Malcom MacLeod) who have been patiently putting out research and tools to support the transparency and reproducibility of pre-clinical research.

I think the one thing that we can all immediately do to improve our science is to improve our understanding of statistics: our psychologist colleagues have assembled great new tool kits where they explain why it is so important to think about the power of an experiment and to report and think about effect sizes. I particularly recommend this course: https://www.coursera.org/learn/statistical-inferences, and the various tools other provided by Daniel Lakens: https://lakens.github.io/statistical_inferences/.

**[KS]:** Good science is not likely when people are under too much pressure to achieve particular results. Putting staff under unreasonable pressure causes problems ranging from wishful thinking in interpretation of data and selective presentation of data, through to complete fabrication. Researchers should love what they are doing and be curious, not desperate. We need to be truth-driven rather than publication-driven, and this is difficult with the current structure of science funding. It is also important to have central storage of research results that are accessible for analysis by a number of people in the group.

Kate, as a Research Integrity Advisor, what practices and values are important to support university-wide integrity?

**[KS]:** Institutions need to be publicly seen to take action on research fraud. The University of Queensland has been ahead of the pack on this issue; a UQ internal investigation led on to Australia’s first criminal conviction for research fraud in 2016. This sends a strong public message. Although this was handled well by our university, we still need an independent national body to oversee investigations of misconduct, as not all institutions may want to air their dirty laundry. As well as our central research integrity office, UQ operates a network of research integrity advisors, including myself. We provide peer advice to concerned staff members on how to address problems including authorship, conflict of interest, and misconduct, with important issues referred to the integrity office. Apart from obvious fraud, research integrity should also be about promoting good rather than slapdash science. These issues are more challenging, requiring training as well as structural change to reward quality and reliability in research rather than number of papers and publication in flashy journals.

As a reviewer, how do you support research integrity?

**[FD]:** My one criterion when I review is whether the work is methodologically well executed. I always support those manuscripts which are, whether their findings confirm or refute the null hypothesis.

**[KS]:** Reviewing a really substantial paper takes at least a day. I don’t accept reviews unless I have time to do them properly. The result of unreasonable expectations in research output is that too many are seeking to publish low quality data. We need to review thoroughly and advise authors on how to improve their submission. I find it surprising that I have to ask so many authors to provide basic information for judging data quality—define at what level replication is done, show all data, define the error bars etc. To avoid publication of fraudulent work, reviewers should not be scared to ask to see the primary data, even in order to put in their first review. Authors should be able to quickly supply primary data. It would be good if journals made it clear to reviewers that they can request primary data.

*This interview was conducted by Zhijuan Qiu for Peer Review Week 2022*

